# Aging, Work, and Care: Typologies of Older Employed Caregivers and Their Well-Being

**DOI:** 10.1093/geroni/igaf122.1074

**Published:** 2025-12-31

**Authors:** Christina Matz

**Affiliations:** Boston College, Chestnut Hill, Massachusetts, United States

## Abstract

As the U.S. population ages, the intersection of work and caregiving is becoming increasingly critical. By 2030, nearly 20% of Americans will be 65 or older, and older adults now represent a rapidly growing segment of the labor force. At the same time, over 53 million Americans provide unpaid care to loved ones, with 61% of caregivers balancing employment and caregiving responsibilities. However, little research has examined the diversity of caregiving experiences among older workers. This study analyzes data from the 2018 Health and Retirement Study (HRS) to identify distinct caregiving typologies among workers aged 50 and older. Using cluster analysis, we identify six caregiving typologies: (1) Non-Caregivers, (2) Grandchild Caregivers, (3) Spousal Caregivers, (4) Parental Caregivers, (5) Financial Caregivers to Parents, and (6) Financial Caregivers to Children. Findings reveal significant differences in sociodemographic characteristics, health outcomes, and well-being indicators across typologies. Notably, spousal caregivers experience the highest levels of stress and depressive symptoms, while grandchild and parental caregivers demonstrate resilience despite caregiving demands. These insights have substantial implications for employers seeking to support caregiving employees. Tailored workplace policies—including flexible work arrangements, mental health support, and financial planning resources—can help mitigate caregiving-related stress. Future research should explore the long-term impacts of caregiving on career trajectories and financial security. By recognizing the varied experiences of working caregivers, policymakers and employers can foster more inclusive workplaces that accommodate the needs of an aging workforce.

